# Effects of anti-RANKL, zoledronate, or combination therapy in a mouse model of fibrous dysplasia: a preclinical study

**DOI:** 10.1093/jbmr/zjag037

**Published:** 2026-02-24

**Authors:** Biagio Palmisano, Chiara Tavanti, Giorgia Farinacci, Alessandro Corsi, Marta Serafini, Natasha M Appelman-Dijkstra, Mara Riminucci

**Affiliations:** Department of Molecular Medicine, Sapienza University of Rome, 00161 Rome, Italy; Institute for Complex Systems, National Research Council, 00185 Rome, Italy; Department of Molecular Medicine, Sapienza University of Rome, 00161 Rome, Italy; Department of Experimental Medicine, Sapienza University of Rome, 00161 Rome, Italy; Department of Molecular Medicine, Sapienza University of Rome, 00161 Rome, Italy; Tettamanti Center, Fondazione IRCCS San Gerardo dei Tintori, 20900 Monza, Italy; School of Medicine and Surgery, University of Milano-Bicocca, 20900 Monza, Italy; Department of Internal Medicine, Division of Endocrinology, Leiden University Medical Centre, 2333 Leiden, The Netherlands; Department of Molecular Medicine, Sapienza University of Rome, 00161 Rome, Italy

**Keywords:** McCune-Albright syndrome, rare bone disease, bone resorption, anti-resorptive drugs, denosumab, zoledronic acid, bisphosphonates, rebound, bone turnover, fractures

## Abstract

Bone fragility and pain are major clinical issues in fibrous dysplasia (FD) of bone, a genetic disorder characterized by increased bone resorption and lytic lesions. Both bisphosphonates (BPs) and denosumab are currently used to treat FD patients, although important concerns remain unsolved. Bisphosphonates downregulate bone remodeling but their effects on FD lesions and pain are variable. Conversely, denosumab converts FD tissue into mineralized bone and prevents disease progression, but disease rebound occurs upon treatment withdrawal. The combination of these 2 drugs may represent an effective and safe strategy for FD treatment. We used a FD mouse model (EF1α-Gsα^R201C^) to assess whether zoledronate (ZOL) addition to anti-RANKL antibody (αRANKL) treatment could preserve the effects of RANKL inhibition after treatment discontinuation. We show that αRANKL treatment rapidly reduced bone turnover markers (BTMs) and increased bone mass in affected skeletal segments, but FD lesions recurred shortly after discontinuation. Importantly, αRANKL+ZOL combination therapy delayed disease rebound after αRANKL withdrawal, as bone density was preserved, BTM rise was prevented, and no new lesions were observed. Zoledronate monotherapy increased bone density and reduced BTMs but did not fully halt disease progression. Finally, both αRANKL and αRANKL+ZOL treatments reduced fracture incidence and improved pain-like behavior in FD mice. These results demonstrate that combining ZOL with denosumab may effectively treat FD. This strategy could particularly benefit patients with severe, rapidly progressive disease, in which RANKL inhibition would block lesion expansion and reduce bone turnover, while ZOL would slow down the resumption of the disease and the rebound effect.

## Introduction

Inhibition of bone resorption is a widely used medical approach for patients with fibrous dysplasia of bone (FD, OMIM#174800), a skeletal disorder caused by activating mutations of the *GNAS* gene.^1^ Fibrous dysplasia can involve one (monostotic) or multiple (polyostotic) skeletal segments, the latter causing more pronounced clinical complications for which pharmacological treatments are needed. The rationale for using anti-resorptive medications in FD patients stems from the abnormally high bone remodeling at affected skeletal sites, where osteoclast formation and function are driven by *GNAS*-mutated osteoprogenitor cells through RANKL secretion.^2^^,^^3^ Indeed, uncontrolled bone resorption is one of the pathogenic determinants that allow the development and expansion of the FD fibro-osseous tissue, which, in turn, severely disrupts the architecture and mechanical properties of skeletal segments.^1^ In FD patients, this pathological sequence can lead to bone deformity, fracture, and pain. Ideally the anti-resorptive treatment in FD, as in other skeletal disorders with high bone remodeling, should not only halt osteoclast activity but also rescue the tissue pathology, thus providing long-term curative effect and likely relief from pain. Furthermore, the treatment should not interfere with the skeletal growth in young patients neither it should cause undesired effects. Two classes of drugs are currently available that target different points of the molecular and cellular cascades linking osteoclast formation and activation to bone resorption, bisphosphonates (BPs), and denosumab. The former are structurally close analogs of pyrophosphate that bind to hydroxyapatite crystals and induce apoptosis of mature osteoclasts through different molecular mechanisms, depending on the absence or presence of nitrogen in their structure.^4^ In particular, aminobisphosphonates are more potent than other BPs due to their higher affinity to hydroxyapatite.^5^ Denosumab is a humanized antibody that binds to RANKL, promoting the apoptosis of preexisting osteoclasts and inhibiting the generation of new ones.^6^ In FD patients, both types of drugs are able to modulate bone turnover markers (BTMs), improve BMD and alleviate bone pain, although the analgesic function of BPs is not confirmed by all studies.^7^ However, denosumab seems to have a higher ability to induce FD lesion refilling with bone and interfere with the progression of the disease. This effect is supported by imaging assessment and bone biopsy in FD patients^8^ and multiple morphological analysis in FD mice, in which the replacing bone has been shown to be lamellar and hyper-mineralized.^2^^,^^9^^,^^10^ The potential reversion of tissue pathology and the relatively reproducible effect on bone pain make denosumab the most promising medical treatment for FD. However, the disease rebound observed in some patients at treatment discontinuation^11^ favors a long-term administration that may have deleterious consequences on the skeleton, especially in pediatric patients. Discontinuation of denosumab treatment represents an important clinical issue also in osteoporosis, as it leads to a rebound effect involving an increase in bone turnover, loss of bone mass gained during the treatment, and risk of new vertebral fractures.^12^^,^^13^ Randomized clinical trials in osteoporotic patients reported that a timely administration of zoledronate (ZOL), a potent third generation BP, may be efficacious in modulating bone biochemical markers after denosumab withdrawal,^14^^,^^15^ suggesting that a refined therapeutic schedule for FD based on both drugs could be a valid strategy to obtain a curative outcome while overcoming safety concerns. However, preclinical studies comparing the effects of RANKL inhibition, BPs, and combination therapy on FD lesions are still missing. Furthermore, it remains unclear whether or not BPs are able to preserve the histological changes induced by RANKL inhibition.

Using the EF1α-Gsα^R201C^ FD mouse model, we previously defined the minimum dose set of an anti-mouse RANKL monoclonal antibody (αRANKL) leading to radiographic and histological refilling of FD lesions, as well as the time interval required for the disease rebound after treatment discontinuation.^2^^,^^10^^,^^16^ In this study, we asked whether this disease-free interval after αRANKL withdrawal could be extended by the addition of ZOL during the treatment. To address this question, we compared the radiographic, biochemical, and histological features of mice exposed to the combination therapy with those of mice receiving either a continuous treatment with αRANKL or ZOL alone. Furthermore, using previously validated behavioral tests on the same mouse model,^17^ we also compared the effect of the different treatments on pain-like behavior in FD mice.

## Materials and methods

### FD mice and treatment

The study was conducted in compliance with relevant Italian laws and Institutional guidelines, and all procedures were IACUC approved. The FD mouse model was generated previously (EF1α-Gsα^R201C^).^18^ In this study, a total of 43 FD mice at 6 mo of age were selected based on the presence of comparable radiographic skeletal phenotypes and randomly assigned to the different experimental groups. Both female (*n* = 26) and male (*n* = 17) mice were included in the study and, since no difference are known to occur in FD lesion appearance and development between the 2 sexes, data were pooled for some analyses. The anti-mouse RANKL monoclonal antibody (αRANKL, clone IK22/5, BioXcell) used at the dose of 12 mg/kg, zoledronic acid (ZOL, Sigma Aldrich) used at the dose of 0.8 mg/kg and the vehicles (VEH) were injected intraperitoneally. The αRANKL dose was chosen based on our previous preclinical study on the same mouse model, showing that this dose was effective in inhibiting RANKL and modifying the tissue pathology of the disease.^2^ To obtain ZOL dose for mice, we decided to use the 4 mg formulation used in FD patients^19^ as reference. Assuming a reference body weight of 60 kg, the resulting human dose of 0.067 mg/kg was multiplied by 12.3 to obtain the mouse dose.^20^

All mice were weighed at the beginning (T0) and the end (T12) of treatment to monitor their body weight.

Experimental groups are shown in detail in Figure 1, while a concise scheme of the dosing regimens is presented in Table 1. VEH group (*n* = 8 mice) received the IgG control isotype (BioXcell) and PBS as vehicles. ZOL group (*n* = 7 mice), received only 2 doses of ZOL. Mice in the other 3 groups received αRANKL as a loading dose (two injections) in the first week and then 2 additional doses, one in the second and one in the fourth week, respectively, a dose set leading to conversion of FD tissue into bone in our model.^10^ Then, the continuous αRANKL group (αRANKL-C, *n* = 10 mice) received a dose of the antibody every 3 wk until the end of the experiment; the discontinued αRANKL group (αRANKL-D, *n* = 7 mice) did not receive any further doses and underwent a 8-wk follow-up; the combination group (αRANKL+ZOL, *n* = 11 mice) received 2 doses of ZOL, one during and the other after the αRANKL-D treatment.

**Table 1:** Concise scheme of the dosing regimen of all experimental groups of treatment. For a detailed scheme of the treatment schedules, please refer to Figure 1.

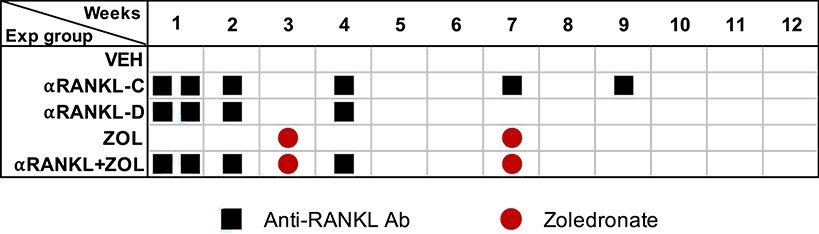

Abbreviations: VEH, vehicle; αRANKL-C, continuous anti-RANKL Ab treatment; αRANKL-D, discontinued anti-RANKL Ab treatment; ZOL, zoledronate treatment; αRANKL+ZOL, anti-RANKL Ab and zoledronate combination treatment.

**Figure 1:**
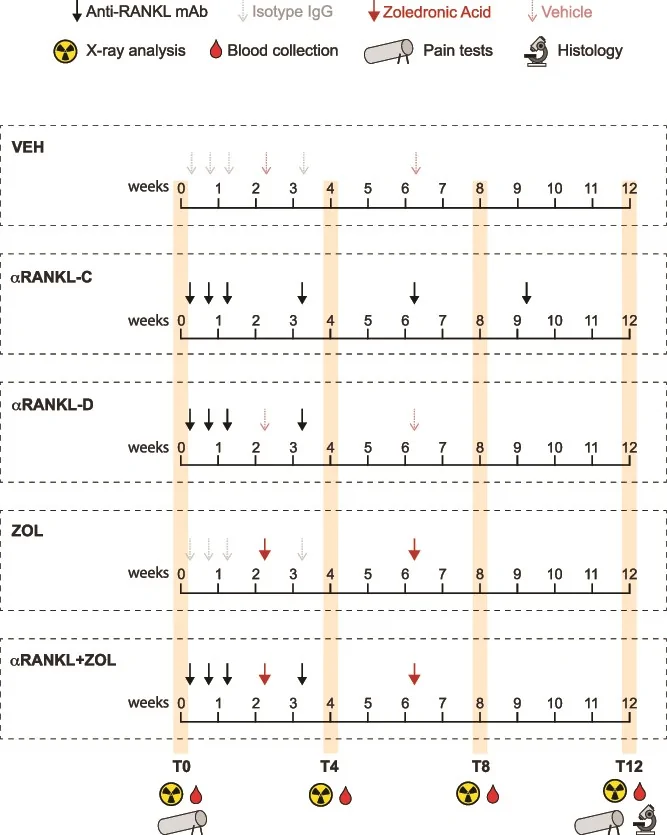
Scheme of the different treatment schedules. All mice were monitored through x-ray imaging and blood analyses at the beginning (T0) and after 4 (T4), 8 (T8), and 12 (T12) weeks of treatment. Pain tests were performed at T0 and before mouse euthanasia, at T12. Histological evaluation of the tissues was performed at T12. VEH, vehicle; αRANKL-C, continuous anti-RANKL Ab treatment; αRANKL-D, discontinued anti-RANKL Ab treatment; ZOL, zoledronate treatment; αRANKL+ZOL, anti-RANKL Ab and zoledronate combination treatment.

All mice were sacrificed 12 wk from the beginning of the experiments by carbon dioxide inhalation.

### Radiographic analysis, quantification of gray values, and disease burden score

Whole body and tail radiographic analyses were performed under general anesthesia before the beginning of treatment (T0) and after 4 (T4), 8 (T8), and 12 wk (T12, Figure 1), using Faxitron MX-20 Specimen Radiography System (Faxitron X-ray Corp.) set at 25 kV for 8 s and 24 kV for 6 s for whole body and tail imaging respectively, with Kodak MINR2000 films. To evaluate changes in the radiodensity of bone segments, radiograms were scanned with an EPSON Perfection V750 PRO Scanner (Epson) at 1200 dpi and saved as JPEG. ROIs were drawn in bone lesions of femurs and caudal vertebrae and radiodensity was evaluated during time by measuring mean gray values (MGVs)^21^ of each lesion using Adobe Photoshop 2024 (Adobe). Fibrous dysplasia lesions were identified as lytic, sclerotic, or mixed areas, often well-circumscribed within the bone segments, with or without a ground-glass appearance. The disease burden score was assessed on radiographic images as described before.^17^ Briefly, a value from 0 to 3 was assigned to each bone segment based on the percentage of the total area occupied by FD lesions (0—no FD lesions; 1—less than 25%; 2—between 25% and 50%; 3—more than 50%), and the final score for each mouse was obtained by summing all individual skeletal segment scores.

### Fracture count

One blinded observer counted the fractures that developed spontaneously in the whole skeleton. We defined the fractures by evidence of discontinuity in the bone structure and/or callus formation with or without bone deformity. In addition to the mean number of fractures per group of treatment, we presented the mean number of new fractures that appeared during the experiment.

### Analysis of BTMs

Blood was collected from the mouse facial vein at the same time points at which radiographic analysis was performed (Figure 1). Blood was allowed to clot for 30 min at room temperature and then centrifuged at 2000 × *g* for 15 min at 4 °C for serum separation. Sera were stored at −80 °C until use. PINP and CTX-I levels were assayed using Rat/Mouse PINP EIA and RatLaps (CTX-I) EIA (Immunodiagnostic System), respectively, following manufacturer indications. Calcium and phosphate levels were analyzed using QuantiChrom kits (BioAssay Systems).

### Histology, histochemistry, and histomorphometry

Mouse skeletal segments were collected after euthanasia at T12 (Figure 1) and fixed with 4% formaldehyde at 4 °C for 48 h. Samples were then decalcified with 0.5 M EDTA at 4 °C for 3-7 d and embedded in paraffin as per standard procedures. Three-μm-thick sections of femurs and lumbar vertebrae were used for histologic and histomorphometric analyses after hematoxylin/eosin (H/E) and sirius red/hematoxylin (SR/H) stains, and tartrate-resistant acid phosphatase (TRAP) histochemistry as previously reported.^22^^,^^23^ Histomorphometric analysis evaluating morphological parameters at lesional sites was performed in a blinded fashion, using ImageJ software and following standard nomenclature.^24^ ROIs were drawn circumscribing FD lesions in femurs and lumbar vertebrae (see dotted areas in Figure 5A). The lesional tissue was defined as areas of mixed fibrous tissue and disorganized bone trabeculae that could be found in trabecular or cortical regions. H/E and SR/H-stained sections were used to measure the volume of trabecular bone per tissue volume (BV/TV), fibrous tissue per tissue volume (Fb.V/TV), and hematopoietic bone marrow per tissue volume (Ma.V/TV). TRAP-stained sections were used to measure osteoclast number per tissue area (N.Oc/TA) and mean osteoclast size (Oc.Size).

Quadriceps and spleens were dissected, weighed, and fixed in 4% formaldehyde at 4 °C for 48 h. After paraffin embedding, transverse sections of the central region of muscles were cut and stained with H/E. The cross-sectional area (CSA) of muscle fibers was quantified using ImageJ. Spleens were embedded in paraffin and sections were obtained and stained with H/E and Perls’ Prussian blue to detect iron deposits. Histomorphometry was performed on stained sections to assess the percentage of red pulp area over total tissue area (RP/TA) and the number of megakaryocytes per tissue area (N.MKs/TA) using ImageJ software.

### Behavioral tests

Burrowing and nesting tests were carried out as previously reported^17^ starting from 6 pm at specified time points (Figure 1). A single investigator performed all procedures in a blinded manner, from animal allocation and handling to the endpoint behavioral measurements.

Burrowing test was carried out using a 20-cm-long tube, with one end of the tube that was sealed with an aluminum lid and the other end that was left open and elevated about 3 cm off the floor. Habituation was carried in group by placing 4-5 mice into a 26 (length) × 23 (width) × 17 (height) cm cage containing the burrow tube filled with food pellets. Forty-eight hours later mice were individually transferred into a cage containing the tube filled with 300 g of pellets for the beginning of the experiment. Burrowing activity was evaluated by weighing the remaining food in the tube after 2 h (2H); at this time, the tube was filled again with 300 g of pellets and evaluation performed after an overnight experiment (ON).

For the nesting test, mice were transferred into individual cages containing 4.5 g of shredded paper as nesting material without any other environmental enrichment. The next morning, the nest was photographed and given a score ranging from 1 to 5 based on the organization and the amount of paper used to build it. In cases where the results did not fit perfectly one of the scores, a half point was either subtracted or added.

### Statistical analysis

Throughout the manuscript, data are presented in 1 of 3 formats: box plots showing all experimental points with horizontal lines indicating the median and vertical lines (whiskers) extending from the minimum to the maximum values; scatter dot plots with individual data points and lines and error bars representing mean ± SD; line graphs connecting mean values across time points.

A repeated measure (RM) one-way ANOVA with Dunnett’s multiple comparison test was used to detect statistical differences among the different time points within each treatment group for MGV, fracture, and BTM analyses. Ordinary one-way ANOVA with Dunnett’s multiple comparison test was used to compare the histological and behavioral endpoints among the different treatments. A 2-way ANOVA mixed-effects model was used when both time and treatment were considered as independent variables, followed by Tukey’s post hoc test for multiple comparisons. In behavioral experiments, a paired *t*-test was used to compare the scores between T0 and T12 within each treatment group. In all experiments a *p*-value value <.05 was considered statistically significant and indicated with one or more asterisks for even smaller *p*-values. Values with .05 ≤ *p* < .1 were considered marginally significant and were reported in the graphs. Statistical analyses and graphing were performed using GraphPad Prism 10 (GraphPad Software).

## Results

### ZOL addition delayed the radiographic recurrence of FD lesions after αRANKL treatment discontinuation

At baseline, the skeletal disease burden score obtained by evaluating the number and extension of FD lesions throughout the mouse skeleton,^17^ was comparable among the experimental groups (Figure S1A). Radiographic monitoring performed at week 4, 8, and 12 on mouse femurs and caudal vertebrae (Figure 1) showed that VEH mice developed new lesions during time and their disease progressed with tendency to skeletal deformities. In the VEH group, no overall differences were observed in the bone radiodensity of the femurs and caudal vertebrae (Figure 2A-D). On the contrary, both αRANKL and ZOL, either alone or in combination, induced changes in regions affected by FD. However, the timeline and magnitude of changes were different depending on the drug and treatment schedule. In femurs, all RANKL-inhibited groups showed a rapid and homogeneous increase in the bone density of lesions, evident during the first radiographic assessment after 4 wk of treatment (Figure 2A and C). Thereafter, in the αRANKL-C group, which continued with one injection of αRANKL every 3 wk, the induced changes in the bone segments were maintained throughout the experiment with no appearance of new lesions (Figure 2A and C). In contrast, in the αRANKL-D group, in which treatment was stopped after 3 wk, lytic lesions recurred, and the typical ground glass appearance was revealed by the imaging performed at week 8. (Figure 2A and C). However, this was not observed if ZOL was added to this αRANKL schedule. In the combined treatment group (αRANKL+ZOL), the lesion opacity was preserved throughout the experiment and a general increase in radiodensity was also observed in the skeletal segments (Figure 2A and C). Overall, the radiological effect of the combined treatment was comparable to that provided by the αRANKL-C schedule (Figure 2C). In mice that received ZOL alone, radiographic changes were also observed albeit at a slower pace compared to the RANKL-inhibited mice. However, since they continued throughout the experiment, at week 12, the gain in radiodensity was comparable to the other treated groups, although small lytic areas persisted creating a non-homogeneous appearance (Figure 2A and C).

**Figure 2:**
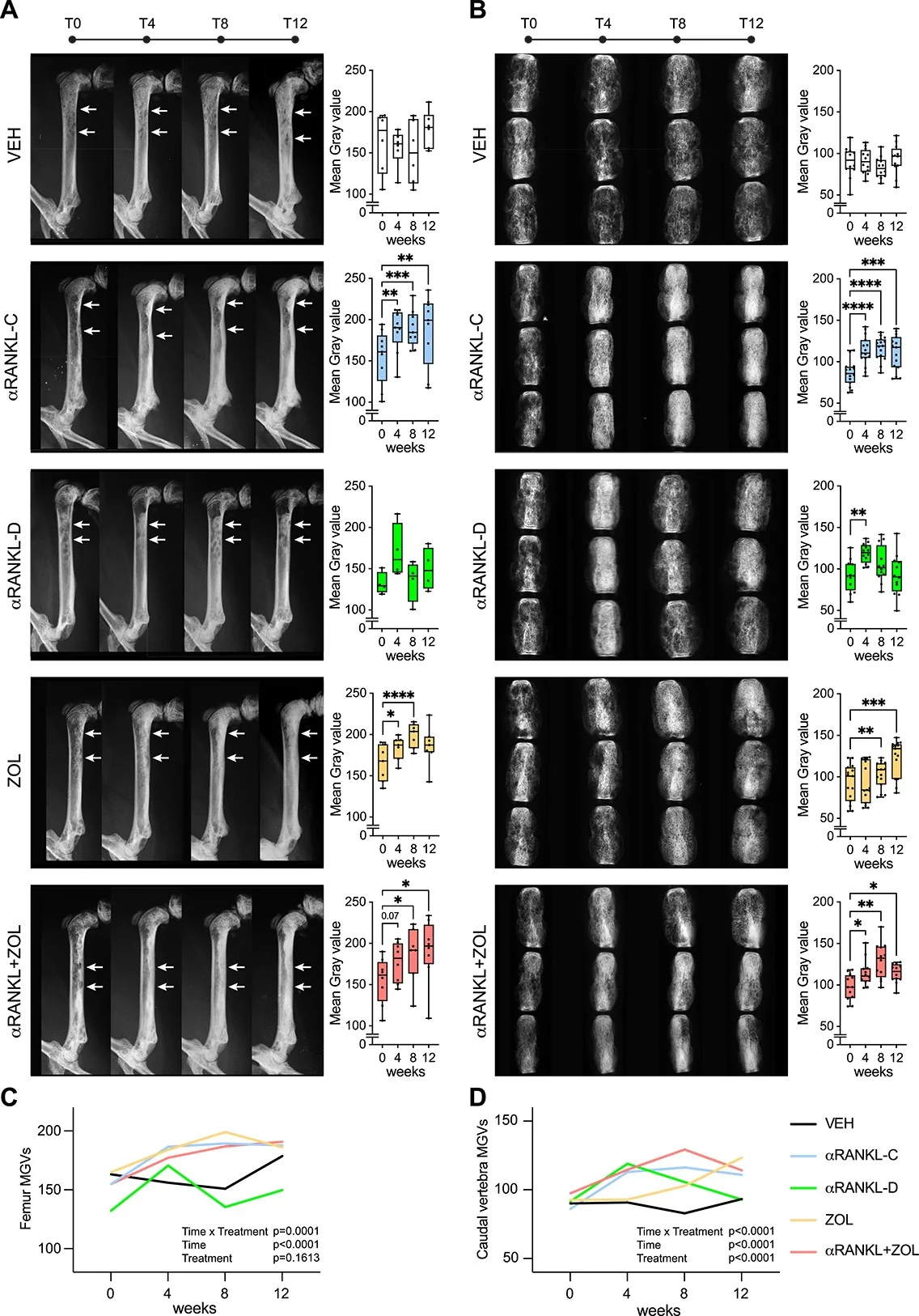
Radiographic effects of the different treatments on EF1α-Gsα^R201C^ mice. (A and B) Radiographic analysis of the same femurs (A) and same caudal vertebrae (B) at different time points with radiodensity quantifications by mean gray values (MGVs). Data are presented as box plots showing all the experimental points. Horizontal line within the box indicates the median. T4, T8, and T12 means were compared to T0 by RM one-way ANOVA Dunnett’s multiple comparison test. (C and D) Grouped analysis of femoral (C) and vertebral (D) radiodensities during time in all experimental groups. *p*-values from the 2-way ANOVA analysis are reported in the graph. In femur analyses, the following sample size are considered per time point: VEH, *n* = 6 femurs from 4 animals; αRANKL-C, *n* = 9 femurs from 5 animals; αRANKL-D, *n* = 4 femurs from 3 animals; ZOL, *n* = 7 femurs from 4 animals; and αRANKL+ZOL, *n* = 8 femurs from 5 animals. In caudal vertebra analyses, the following sample size are considered per time point: VEH, *n* = 12 vertebrae from 4 animals; αRANKL-C, *n* = 15 vertebrae from 5 animals; αRANKL-D, *n* = 12 vertebrae from 4 animals; ZOL, *n* = 15 vertebrae from 5 animals; and αRANKL+ZOL, *n* = 12 vertebrae from 4 animals. In all panels ^*^p < .05, ^**^p < .01, ^***^p < .001, ^****^p < .0001; *p*-value ≤0.1 is reported in the graphs.

In the tail vertebrae, the radiographic results were similar to those of the femurs (Figure 2B). Again, an increase in lesional opacity was seen 4 wk after RANKL inhibition was started and this was maintained throughout the active αRANKL treatment period. This treatment also prevented the abnormal enlargement of the tail vertebrae typically observed in untreated FD mice. As in the femurs, lesion recurrence with a ground glass appearance was detected in the αRANKL-D group at week 8 (Figure 2B and D). The addition of ZOL maintained an overall higher bone density compared to αRANKL-D treated mice (Figure 2D) and prevented the relapse of the disease after αRANKL discontinuation (Figure 2B and D). In mice that received ZOL alone, vertebral radiodensity was markedly increased but the progression of the disease was revealed by ground glass changes and vertebral enlargement (Figure 2B and D).

A 2-way ANOVA was performed to analyze the effect of time and treatment on the MGVs. The analysis revealed a significant interaction between the effects of time and treatment in both femoral and vertebral MGVs (Figure 2C and D). Simple main effects analysis showed that time but not treatment had a statistically significant effect on femur MGVs (Figure 2C), whereas both time and treatment had a statistically significant effect on the density of caudal vertebrae (Figure 2D).

### ZOL addition to αRANKL treatment reduced the incidence of fractures in FD mice

EF1α-Gsα^R201C^ mice develop spontaneous fractures during time; therefore, we decided to analyze the effect of the different treatments on this disease’s complication. Radiographic analyses revealed that most FD mice showed at least one fracture at the beginning of treatment (Figure S1B). Most of the fractures were localized in the tail (92% of total fractures, Figure 3A). We also observed fractures in long bones (7.2%) but rarely in the spine (0.8%). Interestingly, fracture number was positively correlated to the skeletal disease burden score (Figure S1C), suggesting that worsening of disease influenced fracture appearance. During the treatment, the VEH group had an increase in the number of new fractures, although comparisons among time points did not reach statistical significance (Figure 3B). The groups of mice treated with αRANKL-D or ZOL alone showed a marked and statistically significant increase in the number of total fractures at the end of the experiment (Figure 3B). In contrast, mice from the αRANKL-C and αRANKL+ZOL groups showed stabilization of the number of total fractures during the experiment (Figure 3B). We performed a 2-way ANOVA with multiple comparison test to assess potential differences among groups at the different time points. Although a difference in the mean number of fractures was observed among groups at baseline conditions (T0), it resulted not significant at the Tukey’s post hoc test (Figure 3C). Significant differences were observed at T12 between ZOL and αRANKL+ZOL groups (Figure 3C), further confirming the higher efficacy of the combined treatment compared to ZOL monotherapy. Moreover, within αRANKL-D and ZOL groups, significant differences were found in the comparisons T0-T12 and T4-T12 in both groups of treatment (Figure 3C). Accordingly, the analysis of new fractures developed after the beginning of treatment, revealed that while in αRANKL-D and ZOL groups an average of 2 new fractures had developed, in αRANKL-C and αRANKL+ZOL groups, the number of new fractures at week 12 was low and close to 0 (Figure 3D).

**Figure 3:**
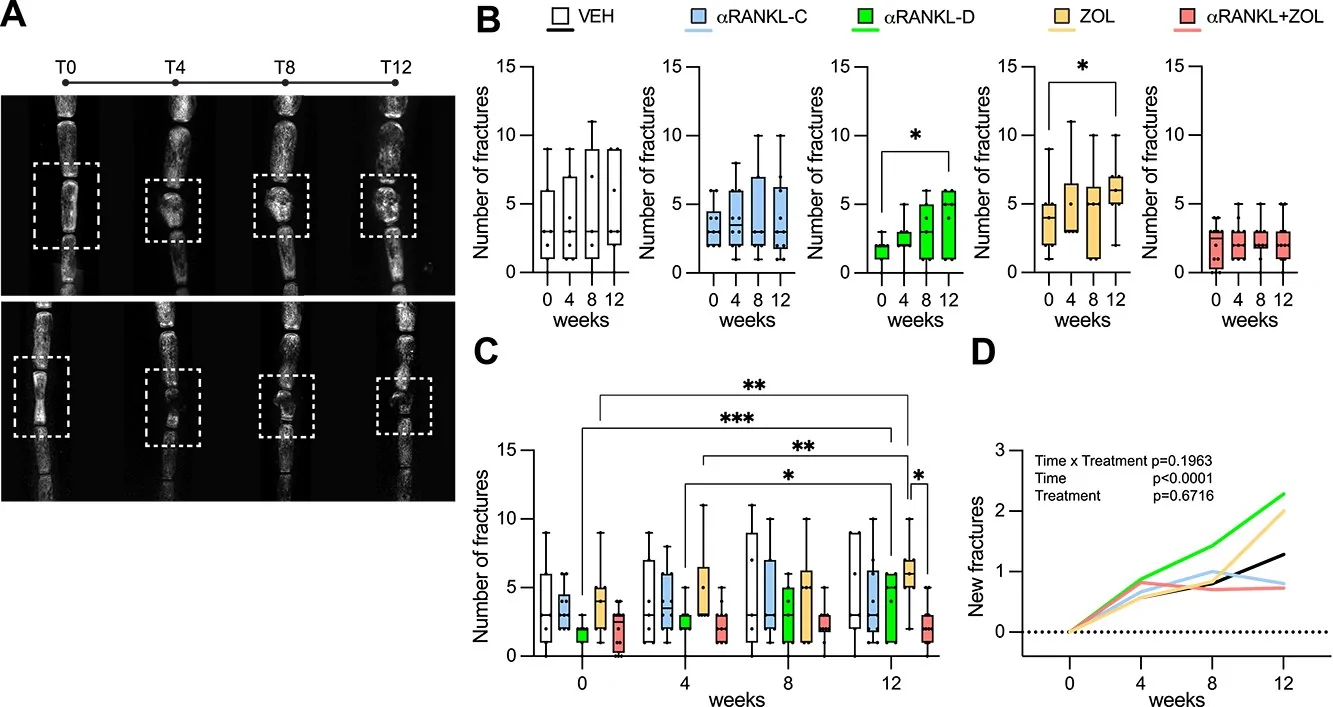
Fracture assessment in EF1α-Gsα^R201C^ mice following the different treatments. (A) Representative radiographs of the evolution of spontaneous vertebral fractures occurring in the caudal vertebrae of FD mice from αRANKL+ZOL (top panel) and VEH (bottom panel) groups. (B) Average number of fractures during time in each treatment regimen. Statistical analysis was performed with one-way ANOVA with post hoc multiple comparison test. T4, T8, and T12 means were compared to T0. (C) Grouped analysis of the average number of fractures in the experimental groups at all time points. Statistical analysis was performed with two-way ANOVA with Tukey’s multiple comparison test. Significant comparisons are reported in the graph. Data are presented as box plots showing all the experimental points. Horizontal line within the box indicates the median. (D) Analysis of the average number of new fractures after the beginning of treatment in each experimental group. *p*-values from the 2-way ANOVA analysis are reported in the graph. In all panels ^*^p < .05, ^**^p < .01, ^***^p < .001, and the following sample size are considered: VEH *n* = 8 mice, ZOL *n* = 7 mice, αRANKL-C *n* = 10 mice, αRANKL-D *n* = 7 mice, and αRANKL+ZOL *n* = 11 mice.

### The addition of ZOL to αRANKL treatment prevented BTM rebound in mice after αRANKL discontinuation

To assess the effect of the different treatments on BTMs, we analyzed the serum levels of CTX-I and PINP, which reflect bone resorption and bone formation activity, respectively. In mice receiving VEH, CTX-I levels tended to increase during the treatment (Figure 4A and B), while no changes in PINP levels were observed during time (Figure 4C and D). In all mice receiving αRANKL, either alone or in combination with ZOL, CTX-I, and PINP were markedly reduced at week 4 compared to baseline levels (Figure 4A-D). In mice treated with αRANKL-C both CTX-I and PINP remained lower than baseline but did not decrease any further after 4 wk (Figure 4A-D). In the αRANKL-D group, BTMs levels started to rise after treatment cessation above baseline values (Figure 4A-D). The observed rise was in parallel to the recurrence of lytic lesions and reached the values of VEH mice at week 12 (Figure 4A-D). Interestingly, mice receiving the αRANKL+ZOL combined treatment, showed BTM changes similar to those of the αRANKL-C group, meaning a steep decrease in 4 wk and plateau phase thereafter (Figure 4A-D), and showed the lowest CTX-I level at all time points (Figure 4B). In mice receiving ZOL alone, BTMs slowly declined during the treatment, resulting lower than VEH treated mice and comparable to the αRANKL-C and αRANKL+ZOL groups at week 12 (Figure 4A-D). Two-way ANOVA analyses performed on CTX-I and PINP grouped data, confirmed the observed synergic effect of time and treatment, as the interaction between the 2 variables was statistically significant in both analyses (Figure 4B and D). The calcium levels did not show significant differences during time in each treatment group (Figure 4E), while slight differences could be observed among the treatment groups (Figure 4F), likely determined by different starting calcium levels. Phosphate levels were overall unchanged during time and among treatments (Figure 4G and H), albeit an increase was noted at week 12 in all treatment groups (Figure 4G and H).

**Figure 4:**
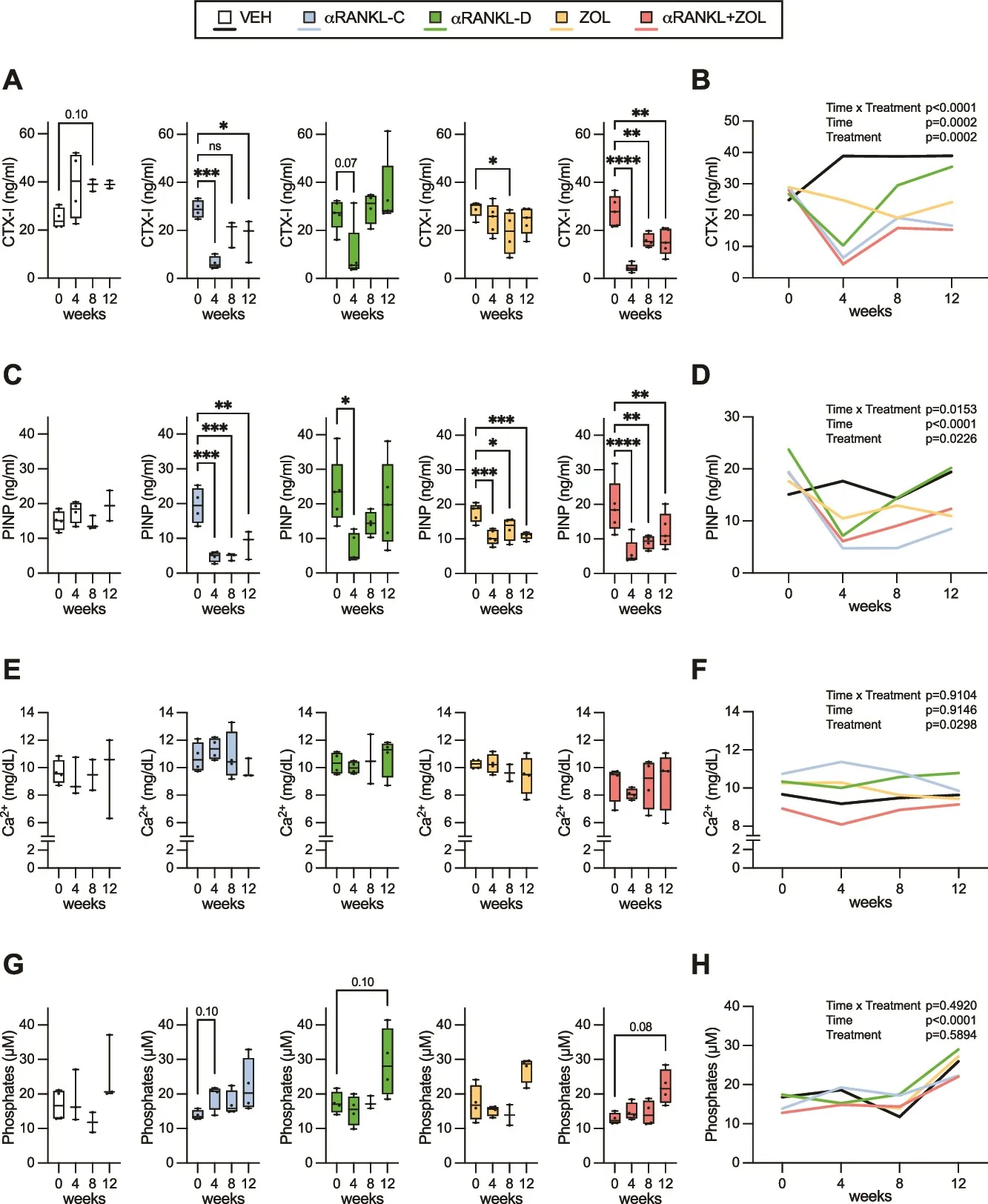
Bone turnover marker analysis at different time points in all treatment schedules. (A) CTX-I levels in each treatment. (B) Grouped analysis of CTX-I levels during time in all experimental groups. (C) PINP levels in each treatment. (D) Grouped analysis of PINP levels during time in all experimental groups. (E) Calcium levels in each treatment. (F) Grouped analysis of calcium levels during time in all experimental groups. (G) Phosphate levels in each treatment. (H) Grouped analysis of phosphate levels during time in all experimental groups. In (A, C, E, and G), data are presented as box plots showing all the experimental points; horizontal line within the box indicates the median. Statistical analysis was performed with RM one-way ANOVA Dunnet’s multiple comparison test, and T4, T8, and T12 means were compared to T0. In (B, D, F, and H), 2-way ANOVA analysis was performed, and *p*-values are reported in the graph. In all panels ^*^p < .05, ^**^p < .01, ^***^p < .001, ^****^p < .0001; *p*-value ≤0.1 is reported in the graphs.

### Combination of ZOL and αRANKL partially improved FD tissue pathology

All mice were sacrificed at T12 and histological and histomorphometric analyses were performed on femurs and lumbar vertebrae (Figures 5 and S2). The VEH group showed all the pathological features of FD, with fibro-osseous lesions occupying the medullary cavity of affected segments (Figures 5A-C and S2A-C). Bone fraction was composed by a mixed woven and lamellar structure (Figure 5A), and the fibrous tissue was accompanied by numerous osteoclasts (Figures 5A and D and S2A and D). An increase in intralesional bone with a lamellar structure was observed in the αRANKL-C group (Figures 5A and B and S2A and B). As expected, no differences compared to VEH were observed in the bone volume of mice in αRANKL-D group as these mice had already lost the bone mass that was previously achieved during the 3-wk treatment course (Figures 5A and B and S2A and B). A significant increase in bone mass compared to VEH was also observed in the group receiving the combination of αRANKL+ZOL and in the ZOL group (Figures 5A and B and S2A and B). The presence of fibrotic marrow was markedly reduced in the αRANKL-C group (Figures 5A and C and S2A and C) as this was the only treatment able to significantly reduce the number of osteoclasts (Figures 5A and D and S2A and D). Interestingly, histomorphometric analysis performed on areas previously occupied by FD lesions revealed that in this experimental group, an important fraction of marrow spaces among the newly formed bone was occupied by normal bone marrow filled with hematopoietic cells (Figures 5A and E and S2A and E). In contrast, large lytic areas filled by fibrous tissue and abundant osteoclasts were detected in mice exposed to the αRANKL-D schedule comparable to VEH group (Figures 5A, C, and  D and S2A, C, and D) and consistent with the radiographic recurrence of the disease after αRANKL discontinuation. Areas of fibrosis were also observed in both αRANKL+ZOL and ZOL groups but these were smaller compared to VEH (Figure 5A and C and S2A and C). In αRANKL+ZOL and ZOL groups, numerous and giant osteoclasts were observed (Figures 5A and S2F and G). As in the αRANKL-C group, a few mice receiving αRANKL+ZOL and ZOL showed small areas of hematopoietic tissue, although only in femurs (Figure 5A and E and S2A and E).

**Figure 5:**
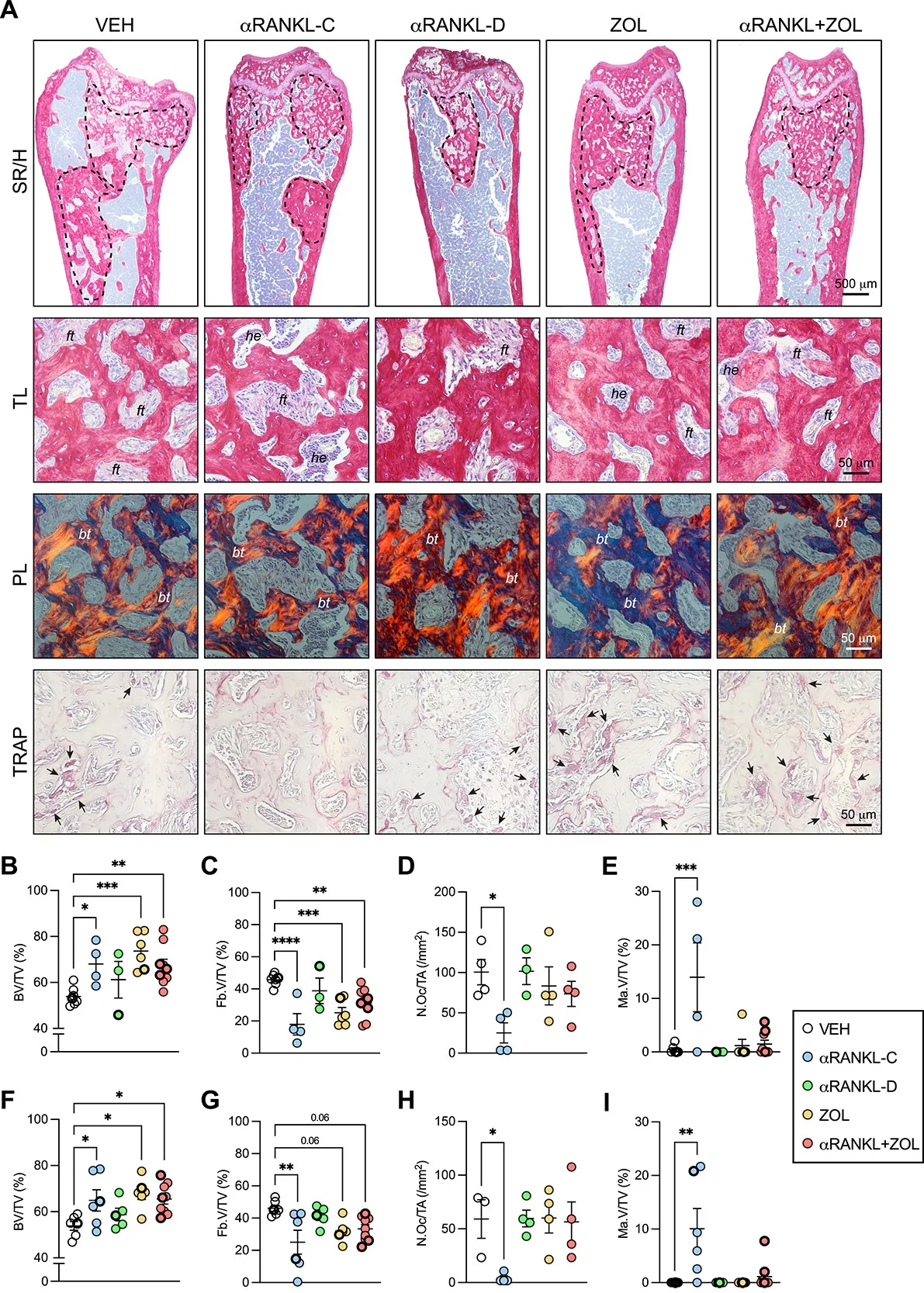
Histological and histomorphometric analyses of femurs in treated EF1α-Gsα^R201C^ mice. (A) Representative sirius red/hematoxylin (SR/H)- and TRAP-stained sections of femurs with FD bone fibro-osseous lesions (dotted areas). Higher magnifications SR/H pictures are imaged with transmitted (TL) and polarized light (PL) microscopy. *ft* = fibrous tissue; *he* = hematopoietic marrow; *bt* = bone trabeculae. TRAP pictures show red-stained osteoclasts (arrows). (B-E) Histomorphometric analysis of intralesional bone volume (B), fibrous tissue volume (C), osteoclast number (D), and hematopoietic marrow volume (E) in femurs. BV/TV, bone volume per tissue volume; Fb.V/TV, fibrous tissue volume per tissue volume; N.Oc/TA, number of osteoclasts per tissue area; Ma.V/TV, hematopoietic bone marrow volume per tissue volume. Data are presented as scatter dot plot, where each dot represents a data point and lines represent mean with SD. Statistical analysis was performed using one-way ANOVA with Dunnett’s multiple comparison test, where mean values from each experimental group is compared to VEH (control) group. ^*^p < .05, ^**^p < .01, ^***^p < .001, ^****^p < .0001; *p*-value ≤0.1 is reported in the graphs. Differential labeling was used in the figure panels to distinguish data from female and male mice (ie, males are represented with a thicker symbol border).

### RANKL inhibition was effective on mouse pain-like behavior

To evaluate the effect of the different treatments on mouse pain-like behavior, we performed the burrowing and nesting tests. Specifically, for each mouse we assessed the behavioral scores at baseline and immediately before the sacrifice (T12, Figure 1).

In the VEH group, the burrowing and nesting behavior during the 12-wk experiment declined (Figure 6A, B, D, and  E), as test results worsened in most of the mice. In particular, the nesting behavior in VEH mice at T12 resulted significantly decreased compared to T0 by either paired *t*-test and 2-way ANOVA (Figure 6D and E). In mice receiving αRANKL, either alone or in combination with ZOL, a consistent amelioration of the pain-like behavior was observed (Figure 6A-F). Burrowing behavior in αRANKL-D mice at T12 resulted significantly increased compared to T0 by paired *t*-test (Figure 6A), and delta change results of the same test revealed a significant increase in αRANKL-D compared to VEH (Figure 6C). Similar results were observed in αRANKL-C group, although T0 vs T12 difference and delta change results compared to VEH did not reach statistical significance (Figure 6A-C). In the group that received αRANKL+ZOL combination therapy, the burrowing and, in particular, the nesting capacity were considerably improved with delta changes resulting significantly increased than VEH (Figure 6A-F). In ZOL treated mice, a worsening of mouse behavior was observed with time, and the test scores were comparable to VEH suggesting that the ameliorating effect of the combination therapy was specifically due to αRANKL (Figure 6A-F).

**Figure 6:**
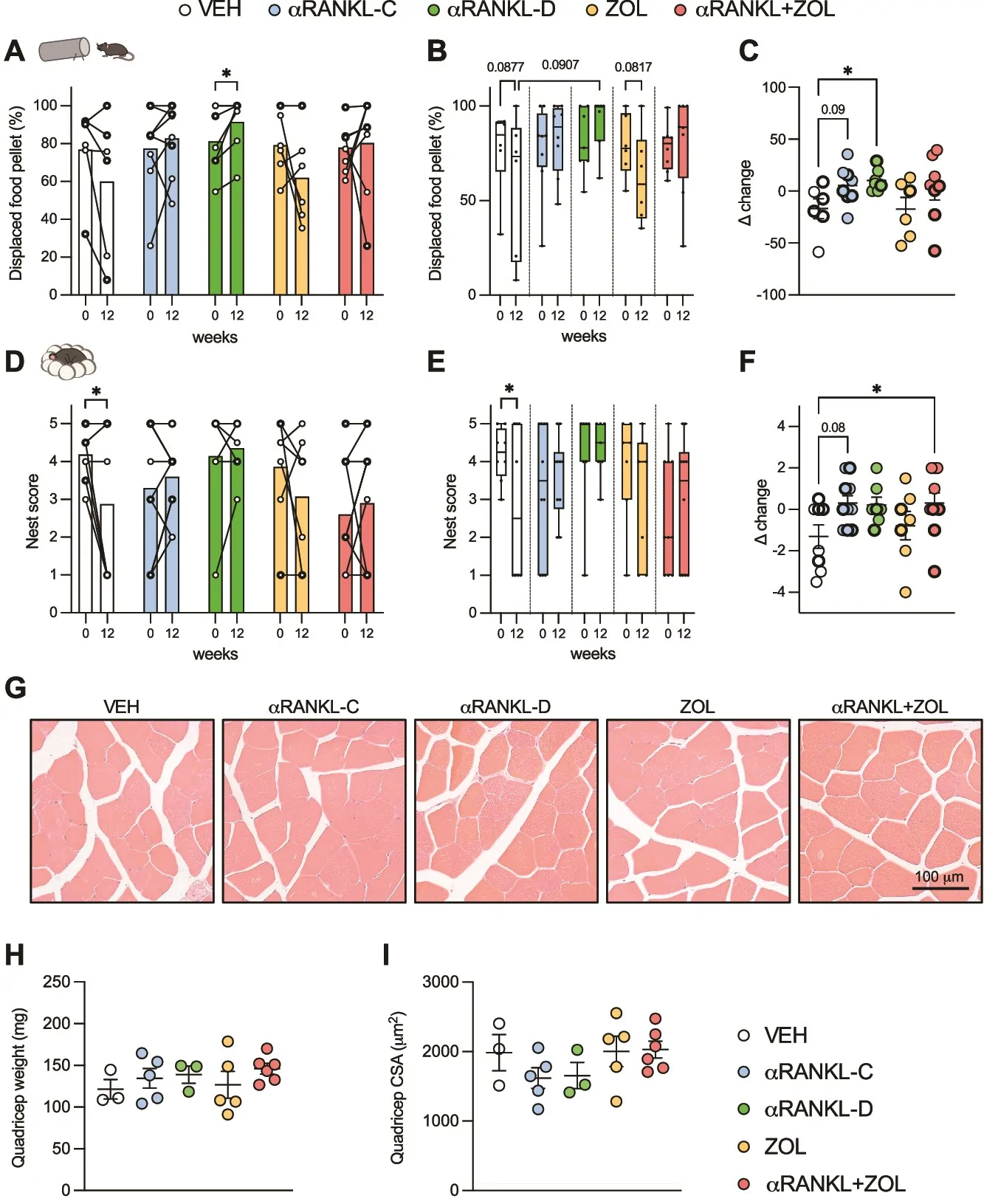
Assessment of pain-like behavior in treated EF1α-Gsα^R201C^ mice. (A) Burrowing test results obtained before (T0) and at the end (T12) of treatment in the different experimental groups showing the behavior of each mouse during time. Each dot represents a mouse and column bars show the mean. Statistical analysis performed with paired *t*-test. (B) The results reported in (A) are showed as mean with SD and analyzed with two-way ANOVA with Tukey’s multiple comparison test. (C) Delta change results calculated by subtracting T0 from T12 burrowing scores and presented as scatter dot plot, where lines represent mean with SD. Data above 0 indicates improvement in the behavioral result, while those below 0 reflect a behavioral worsening during the treatment. Statistical analysis performed with one-way ANOVA. (D) Nesting test results obtained before (T0) and at the end of treatment (T12) in the different experimental groups. Each dot represents a mouse and column bars show the mean. Statistical analysis performed with paired *t*-test. (E) The results reported in (D) are shown as mean with SD and analyzed with 2-way ANOVA with Tukey’s multiple comparison test. (F) Delta change results calculated by subtracting T0 from T12 nesting scores and presented as scatter dot plot, where lines represent mean with SD. Statistical analysis performed with one-way ANOVA. ^*^p < .05; *p*-value ≤0.1 is reported in the graphs. (G) Representative histological hematoxylin/eosin-stained sections of muscle fibers in mice at the end of treatments. (H) Weight of quadriceps dissected from female mice at the end of treatments. (I) Histomorphometric analysis of muscle fiber cross sectional area (CSA) from female mice only. (H) and (I) data are presented as scatter dot plot, where lines represent mean with SD. In all graphs, each dot represents a data point. In (A-F), differential labeling was used to distinguish data from female and male mice (ie, males are represented with a thicker symbol border).

Of note, mice with severely reduced burrowing scores in basal conditions, that is, burrowing less than 25% of the pellet food, did not show any amelioration, independent of the type of treatment (data not shown).

### Inhibition of bone resorption did not affect muscle fiber size

It has been recently suggested that RANKL inhibition may be beneficial for skeletal muscle function.^25^ To understand if the improvement in the behavioral capacities seen in αRANKL-treated mice could be explained, at least in part, by improved muscular parameters, we performed a morphological evaluation of quadriceps (Figure 6G). Muscle weight was similar among the different experimental groups at the end of treatment (Figure 6H). Histomorphometric analysis of the CSA of the quadriceps revealed no differences among the groups, although a trend toward an increase of muscle fibers area was observed in αRANKL+ZOL-treated animals (Figure 6I).

### ZOL administration induced splenomegaly and accumulation of megakaryocytes in spleens

We did not observe changes in the body weight of mice in the different experimental groups, except for ZOL-treated mice, in which a significant increase was noticed (Fig. S3). At the time of the sacrifice, necroscopy showed that some mice had larger spleens (Figure 7A). In particular, the spleens of mice receiving ZOL, either alone or in combination with αRANKL, were larger and heavier than those of VEH treated animals (Figure 7A and B). Histological and histomorphometric analyses did not reveal differences in the iron deposits or amount of red pulp per tissue area among the different experimental groups (Figure 7C and D), thus indicating the expansion of the entire splenic parenchyma rather than an unbalanced red/white pulp ratio. Interestingly, however, an increase in the number of megakaryocytes often with clear intranuclear vacuoles was observed in the ZOL-treated animals compared to VEH group (Figure 7E and F).

**Figure 7:**
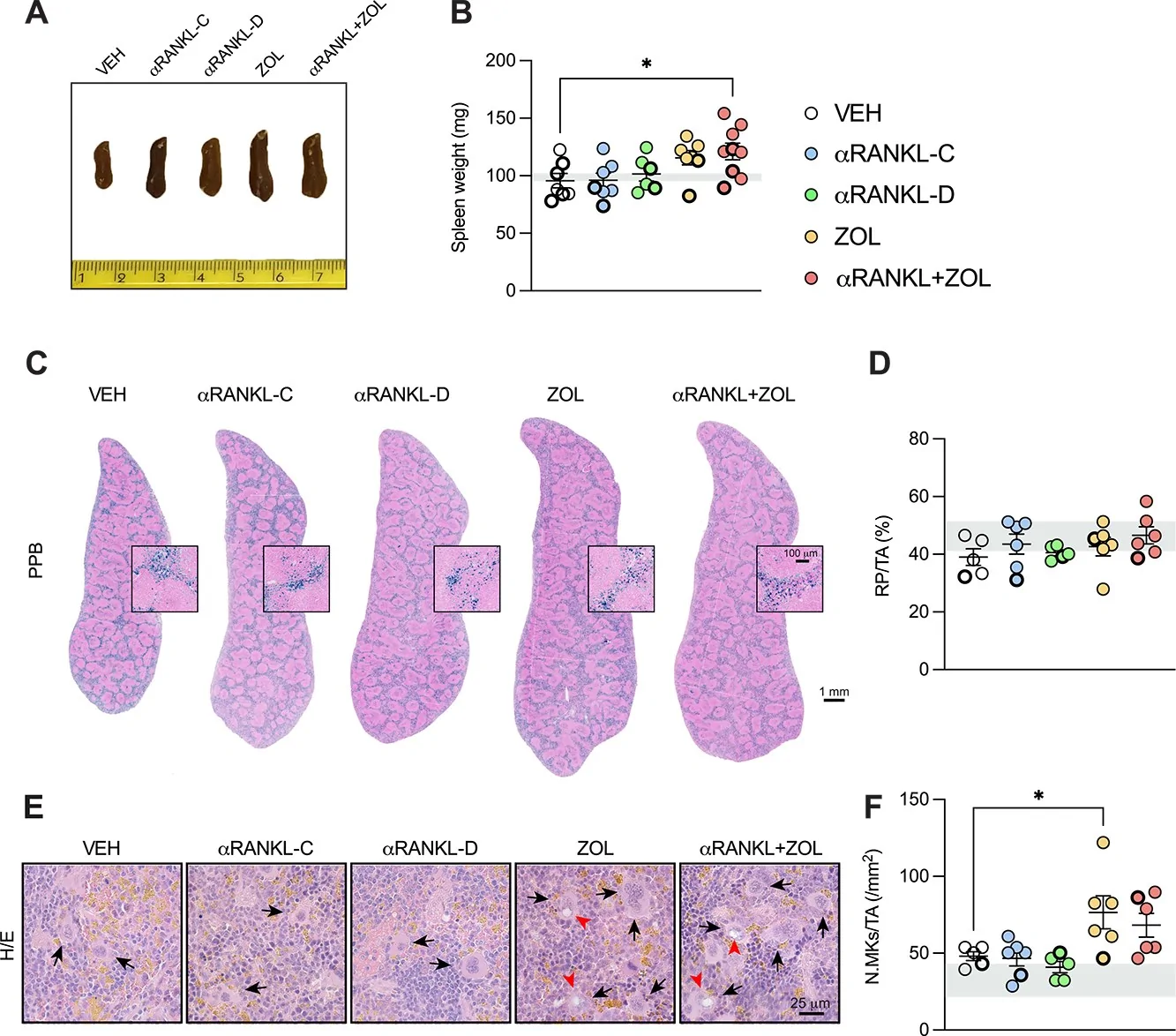
Assessment of spleen morphology in EF1α-Gsα^R201C^ mice at the end of the different treatments. (A) Pictures of representative spleens from each group of treatment. (B) Weight of spleen dissected from the mice at the end of treatments. (C) Representative pictures of spleen sections stained with Perls’ Prussian blue (PPB) to detect iron deposits (blue-stained). (D) Histomorphometric evaluation of red pulp over total tissue area (RP/TA). (E) Picture of H/E-stained spleen sections showing megakaryocytes (arrows), some of them with intranuclear vacuoles (red arrowheads). (F) Histomorphometric evaluation of megakaryocyte number over total tissue area (N.MKs/TA). Gray bands in the panels represent WT ranges for each specific parameter. In all graphs, data are presented as scatter dot plot, where each dot represents a data point and lines represent mean with SD. Statistics performed with one-way ANOVA with Dunnett’s multiple comparison test, with ^*^p < .05. Differential labeling was used to distinguish data from female and male mice (ie, males are represented with a thicker symbol border).

## Discussion

Discontinuation of denosumab raises a major clinical issue since post-treatment bone turnover activity is usually exaggeratedly increased compared to baseline and the bone mass gained during the treatment is rapidly lost.^26^ In osteoporotic patients, this rebound effect associates with the risk of new vertebral fractures,^12^^,^^13^ whereas in high bone remodeling diseases, such as FD, it may also be accompanied by severe hypercalcemia.^11^^,^^27^ While theoretically feasible, long-term administration of denosumab is not recommended and strategies to preserve its effect after treatment cessation are highly needed. Clinical studies in osteoporosis suggest that administration of ZOL may be a reasonable approach, at least in patients who received a short-term denosumab treatment^14^^,^^15^ and The European Calcified Tissue Society (ECTS) recently proposed repeated administrations of this BP in patients with elevated BTM levels after denosumab discontinuation.^28^

In order to start collecting proof-of-concept data that this strategy could be also effective in FD, we carried out a preclinical study on the EF1α-Gsα^R201C^ mouse model of the disease in which we compared different treatments including continuous αRANKL, discontinued αRANKL, ZOL, and αRANKL+ZOL combination. In the combined treatment, ZOL was administered during and after the αRANKL administration. The rationale for the addition of ZOL at an early phase during RANKL inhibition was to ensure a proper incorporation into the bone, as BPs are known to bind eroded surfaces.^29^ The later ZOL injection was conceived to increase the amount of drug bound to hydroxyapatite. This second dose of ZOL was provided 3 wk after the end of RANKL inhibition since, based on pilot studies, this is the optimal dosing interval to maintain the effect of αRANKL in long-term treatments in EF1α-Gsα^R201C^ mice.

We show that ZOL was effective in preserving the radiographic improvement of femoral FD lesions for at least 8 wk after the last dose of αRANKL, with an overall level of bone density that was comparable to that of mice in the αRANKL-C group. Of note, a significant increase in the radiodensity of affected segments was also observed in mice receiving ZOL alone, in agreement with previous studies reporting radiological improvement in pediatric FD patients treated with this BP.^30^ However, compared to both the αRANKL-C and the combined groups, bone mass accrual in ZOL receiving mice proceeded slower and led to more marked bone enlargement/deformities. This can be reasonably explained by the different mechanism of action of the two drugs. By completely blocking bone resorption, αRANKL allowed the rapid conversion of the fibrous tissue into bone,^2^^,^^16^ after which pathological bone was no longer formed. In contrast, during ZOL treatment, pathological bone continued to be deposited while its resorption was reduced due to the downregulation of osteoclast function. Furthermore, despite an increase in radiodensity, ZOL alone did not prevent the occurrence of new fractures, mainly occurring in the caudal vertebrae in these mice. In contrast, mice that received the αRANKL+ZOL schedule showed the lowest incidence of fractures, as those in the αRANKL-C group, suggesting that the addition of ZOL may also preserve the beneficial effect of RANKL inhibition on this clinical complication.^31^ Fractures in FD can occur spontaneously and are associated with skeletal burden,^32^ as we also observed in our FD mouse model. This is a consequence of the intense osteoclastogenesis and bone resorption that alters bone architecture, leading to bone deformity and weakness. Our results are in agreement with the clinical evidence that in FD, radiographic amelioration induced by BPs is not predictive of a lower fracture risk^33^ as also observed in osteoporosis.^34^ On the contrary, they suggest that complete inhibition of osteoclastogenesis by αRANKL may be beneficial to prevent the appearance of new spontaneous fractures, although confirming this finding in FD patients would require long term denosumab treatments that are difficult to perform.

At the biochemical level, ZOL prevented the rise of BTMs observed after αRANKL discontinuation, maintaining their levels comparable to those of αRANKL-C mice at all time points. Consistent with reports on FD patients,^35^ a reduction of BTMs was also observed in mice receiving ZOL alone, especially at weeks 8 and 12. However, the difference between the pre- and post-treatment level was smaller than that observed in both the αRANKL-C and the combined groups. Altogether, these data confirm that in FD, αRANKL administration provides a rapid improvement of radiographic features and BTMs and suggest that ZOL may be a good option to maintain these results over time. Since mice were monitored for 8 wk after the last dose of αRANKL, it can be predicted that in FD patients receiving a similar schedule the effect of ZOL should last more than 5 yr.^36^ We should, however, note that the FD mouse model used in this study harbors a ubiquitously expressed *Gsα*^R201C^,^18^ while human lesions are mosaics including both WT and GNAS-mutated cells. It is likely therefore that prevention of rebound could be more difficult in our FD mouse model compared to FD patients, implying an even better response in the latter. Further studies are required to translate this schedule into clinical settings.

We previously showed that a rise in calcium levels in young EF1α-Gsα^R201C^ mice occurred after 14-wk treatment with high cumulative dose of αRANKL.^2^ In this study, in which the total amount of αRANKL and the treatment duration were much lower than in the previous study, we did not observe any case of hypercalcemia. This suggests that hypercalcemia after discontinuation of αRANKL may occur in long duration, and therefore high cumulative dose, treatments, a hypothesis that seems to be corroborated by recent clinical studies.^27^^,^^37^ Furthermore, since our previous study used very young mice,^2^ whereas the current study treated adult mice, the observed hypercalcemia may be age-dependent—consistent with rebound hypercalcemia being more common in young patients following denosumab cessation.^27^^,^^38^

Consistent with the radiographic results, histomorphometric analyses performed at the end of the experiment showed a higher bone mass compared to controls in all the experimental groups, except for mice that underwent a short RANKL inhibition. As previously reported, continuous administration of αRANKL was the only treatment able to reduce significantly the amount of fibrous tissue and restore the hematopoietic microenvironment.^2^ However, a decrease in the volume of fibrous tissue was also observed in mice receiving ZOL alone. The notion that ZOL in FD can partially reduce the fibrous tissue was never reported before and may be taken as further evidence that osteoclast activity plays a major role in the tissue pathology of FD. Typical histological feature of ZOL treatment, either with αRANKL or alone, was the presence of giant osteoclasts, as already noticed in the same transgenic mouse model of FD.^16^ Giant osteoclasts following treatment with nitrogen containing BPs were initially described in postmenopausal women as cells with extended lifespan and poor bone resorbing capacity.^39^ Although we did not investigate the mechanism of formation of giant osteoclasts, the strong decrease in the bone resorption marker CTX-I in the αRANKL+ZOL and ZOL groups confirmed that even in FD giant osteoclasts have a reduced bone resorbing activity and this may contribute to the positive effect on intralesional bone mass exerted by this BP.

To add more clinical perspective to our work, we also performed specific tests to assess mouse pain-like behavior at both the beginning and end of each treatment. We observed that the test scores were improved by αRANKL, either alone or in combination with ZOL. Pain improvement by denosumab was reported in many FD patients,^11^^,^^40^^,^^41^ and it was speculated to be based on the reduction of the acidic environment caused by complete ablation of osteoclasts. This hypothesis is in agreement with our work, as both continuous administration of αRANKL and combination therapy with ZOL induce ameliorating effects on the pain-like behavior of treated mice. However, we also show that behavioral scores were improved in αRANKL-D mice, in which rebound had occurred, osteoclasts had regenerated and FD had already relapsed. This result could be hardly reconciled with a role for the acidic environment in FD pain. Nonetheless, it is plausible that a short RANKL inhibition may still be sufficient to generate an early analgesic effect, as reported in patients with low back pain,^42^ and a longstanding response. Of note, in mice with severe behavioral impairment at baseline, no amelioration was observed at the end of the experiment, regardless of treatment. This finding may explain the absence of significant analgesic effects of denosumab in some FD patients. More important, it suggests that the treatment should be administered soon after the appearance of pain in order to obtain a positive response. As for the ZOL monotherapy, the treatment did not affect the behavioral score as a behavioral deterioration comparable to that of VEH mice was observed in the time frame of the experiment. Data on pain relief by BP treatment in FD patients are controversial.^43^ Pamidronate, a nitrogen-containing BP, has shown good analgesic effect in 8 out of 13 patients with pain, which was maintained 36 mo after the treatment.^44^ However, a double blind RCT with another nitrogen-containing BP, alendronate, showed no significant difference compared to placebo in terms of pain relief.^45^ In a recent study, treatment with ZOL has shown partial or complete relief of bone pain in 8 out of 13 patients.^35^ This suggests that analgesic response to BPs may result from unknown mechanisms and interindividual variability. Since it has been shown that pain in FD does not relate to pathological sensory nerve fiber growth but may rather depend on nociceptive mechanisms activated by inflammatory cytokines or neurotrophic factors released by FD cells,^17^^,^^23^^,^^46^ the variable effects of BPs in FD patients and the lack of efficacy of ZOL in FD mice could reflect the inability of these drugs to modify the cellular and molecular profile of the pathological tissue.

Since recent studies demonstrated that αRANKL can produce beneficial effects on muscle mass and function in mice^25^ (although this is still a controversial issue in humans^47^), we also analyzed skeletal muscle morphology. We did not observe significant differences in quadricep weight, nor in the CSA of muscle fibers among the different groups. A slight tendency to larger muscle fibers was observed in ZOL treated mice, a result already reported in other mouse models of high bone remodeling.^48^ However, ZOL did not ameliorate pain-related behavior, thus ruling out a potential association between muscle structure and function and the results of the behavioral analyses.

Finally, we found that mice receiving ZOL, either in combination with αRANKL or alone, had larger spleens with morphologically abnormal megakaryocytes compared to the other experimental groups. It was previously reported that in mice injected with a nitrogen-containing BP (namely the AHBuBP) the spleen was considerably larger than in mice receiving a chloro-containing BP and controls.^49^ Bisphosphonate-dependent splenomegaly can be ascribed to the expansion of the red pulp with an increased number of megakaryocytes as well as to the development of extramedullary erythropoiesis in parallel with the depletion of bone marrow-resident macrophages.^49^ In addition, it is known that nitrogen-containing BPs accumulate for several days into liver and spleen^50^ after the formation of complexes with calcium and iron.^4^ We observed an increase in megakaryocytes but no difference in the amount of iron in the spleen in our ZOL-treated mice compared to the other experimental groups, thus we did not identify a potential pathogenic mechanism. Further investigation is required to clarify this point. However, it is important to remark that to date, splenomegaly upon treatment with nitrogen containing BPs has been observed only in mice and therefore we cannot exclude that this side effect may be species-specific, resulting, for example, from the presence of physiological hematopoietic activity in the mouse but not human spleen.

In conclusion, we report that in a mouse model of FD, ZOL addition during and after RANKL inhibition was able to preserve the intralesional bone mass gain and to maintain the effect on BTMs and fracture risk for at least 8 wk after αRANKL discontinuation. We also show that ZOL monotherapy was able to increase bone mass but did not prevent the expansion/deformity of affected skeletal segments neither it reduced the incidence of bone fractures and pain.

We acknowledge that this study has some limitations. Specifically, histological endpoints should have been performed at different time points to better evaluate the effects of the combination therapy on the lesional tissue; multiple αRANKL+ZOL combination schedules should have been tested to gain insights into the clinical translatability of the combined approach; the use of adult mice did not address the feasibility of the combination approach in the pediatric FD population, in which prolonged denosumab treatment represents a major concern. However, we believe that, the combination of denosumab with ZOL may be a useful approach in FD patients with a very active and rapidly progressive FD in which a short course of RANKL inhibition may be necessary to block lesion expansion/bone deformities and to relieve bone pain, whereas ZOL may be useful to slow down the resumption of the disease.

## Supplementary Material

4)_Supplementary_material_R2_Clean_zjag037

## Data Availability

The data underlying this article will be shared on reasonable request to the corresponding author.
