# Supplementary material for: Effects of anti-RANKL, zoledronate, or combination therapy in a mouse model of fibrous dysplasia: a preclinical study
Source: J Bone Miner Res. 2026 Feb 24;41(11):1249–64. doi: 10.1093/jbmr/zjag037 (PMC13716912; doi:10.1093/jbmr/zjag037)
Supplement: Supplementary file 1 [file 4)_supplementary_material_r2_clean_zjag037.docx]

**Figure S1**

**Figure S1. Baseline skeletal phenotype of the FD mice randomly assigned to each group of treatment.** A) Skeletal disease burden score assessed through the evaluation of the number and extension of FD lesions by x-ray analyses. B) Mean fracture number evaluated on radiographs. C) Pearson correlation analysis between disease burden and number of fractures.

**Figure S2**

**Figure S2. Histological and histomorphometric analyses of lumbar vertebrae in treated EF1α-Gsα^R201C^ mice.** A) Representative SR/H- and H/E-stained sections of lumbar vertebrae with fibro-osseous lesions. *ft* = fibrous tissue; *he* = hematopoietic marrow. B-E) Histomorphometric analysis of intralesional bone volume (B), fibrous tissue volume (C), osteoclast number (D) and hematopoietic marrow volume (E). F) Histomorphometric evaluation of osteoclast size (Oc. Size) in female mice. Statistical analysis with one-way ANOVA and Dunnett’s multiple comparison test where mean values from each experimental group is compared to VEH (control) group. G) Frequency of distribution of different osteoclast size range. Two-way ANOVA and multiple comparison test. *p<0.05, **p<0.01, ***p<0.001. BV/TV: bone volume per tissue volume. Fb.V/TV: fibrous tissue volume per tissue volume. N.Oc/TA: number of osteoclasts per tissue area. Ma.V/TV: hematopoietic bone marrow volume per tissue volume. Data are presented as scatter dot plot, where each dot represents a data point and lines represent mean with SD. *p<0.05, **p<0.01, ***p<0.001; p-value ≤0.1 is reported in the graphs. Differential labeling was used in the figure panels to distinguish data from female and male mice (i.e. males are represented with a thicker symbol border).

**Figure S3**

**Figure S3. Body weight changes during the treatment in EF1α-Gsα^R201C^ mice.** a) Body weight values at the beginning (0) and the end (12) of treatment. Two-way ANOVA with Tukey’s multiple comparison test. b) Delta change results calculated by subtracting T0 from T12 body weight and presented as scatter plots, in which is shown mean with SD.
